# Association between computed tomography-quantified respiratory muscles and chronic obstructive pulmonary disease: a retrospective study

**DOI:** 10.1186/s12890-024-02955-5

**Published:** 2024-03-21

**Authors:** Ke Wang, Fan Wu, Hua He, Chengyi Hu, Xiaobang Chen, Jinglong Chen, Weitao Cao, Jun Liu, Jun Zhao, Ziwen Zhao, Zhuxiang Zhao

**Affiliations:** 1 Department of Infectious Diseases, Respiratory and Critical Care Medicine, Guangzhou First People’s Hospital, South China University of Technology, Guangzhou, China; 2 Guangzhou Chest Hospital, Guangzhou, China; 3 State Key Laboratory of Respiratory Disease & National Clinical Research Center for Respiratory Disease & National Center for Respiratory Medicine & Guangzhou Institute of Respiratory Health, The First Affiliated Hospital of Guangzhou Medical University, Guangzhou National Laboratory, Guangzhou, China; 4 Department of Geriatrics, National Clinical Key Specialty, Guangzhou First People’s Hospital, South China University of Technology, Guangzhou, China

**Keywords:** COPD, Accessory respiratory muscles, CT, Pectoralis major

## Abstract

**Background:**

This study examined the association between chest muscles and chronic obstructive pulmonary disease (COPD) and the relationship between chest muscle areas and acute exacerbations of COPD (AECOPD).

**Methods:**

There were 168 subjects in the non-COPD group and 101 patients in the COPD group. The respiratory and accessory respiratory muscle areas were obtained using 3D Slicer software to analysis the imaging of  computed tomography (CT). Univariate and multivariate Poisson regressions were used to analyze the number of AECOPD cases during the preceding year. The cutoff value was obtained using a receiver operating characteristic (ROC) curve.

**Results:**

We scanned 6342 subjects records, 269 of which were included in this study. We then measured the following muscle areas (non-COPD group vs. COPD group): pectoralis major (19.06 ± 5.36 cm^2^ vs. 13.25 ± 3.71 cm^2^, *P* < 0.001), pectoralis minor (6.81 ± 2.03 cm^2^ vs. 5.95 ± 1.81 cm^2^, *P* = 0.001), diaphragmatic dome (1.39 ± 0.97 cm^2^ vs. 0.85 ± 0.72 cm^2^, *P* = 0.011), musculus serratus anterior (28.03 ± 14.95 cm^2^ vs.16.76 ± 12.69 cm^2^, *P* < 0.001), intercostal muscle (12.36 ± 6.64 cm^2^ vs. 7.15 ± 5.6 cm^2^, *P* < 0.001), pectoralis subcutaneous fat (25.91 ± 13.23 cm^2^ vs. 18.79 ± 10.81 cm^2^, *P* < 0.001), paravertebral muscle (14.8 ± 4.35 cm^2^ vs. 13.33 ± 4.27 cm^2^, *P* = 0.007), and paravertebral subcutaneous fat (12.57 ± 5.09 cm^2^ vs. 10.14 ± 6.94 cm^2^, *P* = 0.001). The areas under the ROC curve for the pectoralis major, intercostal, and the musculus serratus anterior muscle areas were 81.56%, 73.28%, and 71.56%, respectively. Pectoralis major area was negatively associated with the number of AECOPD during the preceding year after adjustment (relative risk, 0.936; 95% confidence interval, 0.879–0.996; *P* = 0.037).

**Conclusion:**

The pectoralis major muscle area was negative associated with COPD. Moreover, there was a negative correlation between the number of AECOPD during the preceding year and the pectoralis major area.

## Introduction

Chronic obstructive pulmonary disease (COPD) is a common respiratory system disease with progressive development and incompletely reversible airflow limitation [1]. The typical symptoms are cough, sputum, and difficulty breathing, resulting in reduced quality of life and daily activities [2, 3]. The high prevalence, high mortality, and challenges in reversing the progressive airway destruction and worsening dyspnea of COPD have plagued clinicians and scientific researchers.

COPD is a wasting or catabolic disease in patients with a low body mass index (BMI). However, BMI indicates fat content that does not effectively reflect muscle characteristics. A low-fat-free mass index may better indicate COPD wasting. A previous study found that the low-fat mass index obtained from the pectoral muscle area was an independent risk factor for COPD mortality [4]. When the catabolism reaches a certain level, there are pathological changes. Muscle wasting and atrophy are common complications of COPD and can lead to decreased skeletal muscle function and exercise capacity, increased energy consumption, and damage to overall health [1, 5]. Changes in body composition are related to COPD, and researchers have found the incidence of muscle atrophy in COPD patients was 20% [6]. Systemic complications of COPD such as skeletal muscle catabolism further aggravate patients’ respiratory symptoms, limit their mobility, and affect their quality of life, increasing the risk of poor prognosis [7]. Therefore, the acute onset coefficient also increases. As the disease progresses, activities are restricted, and the mechanical ventilation rate, hospitalization rate, and mortality rate of patients experiencing acute exacerbations increase sharply [8, 9]. Therefore, skeletal muscle atrophy is considered to be closely related to the high mortality and poor prognosis of COPD [10].

Clinically, we have found that many patients with normal lung function produce poor imaging of the lungs and disease progression from computed tomography (CT), which is not consistent with the degree of lung function airflow limitation. At present, the diagnosis and evaluation of patients with COPD are carried out using airflow measurements of lung function. Although these measurements are critical, they do not describe the status of the entire patient population and individual patients and may also obscure the underlying COPD pathomorphology. Independent contribution CT is a common imaging technique and is currently a method for evaluating skeletal muscle. The skeletal muscle area measured by CT has been correlated with healthy subjects' total skeletal muscle mass [11]. Therefore, CT has been an effective method to evaluate the skeletal muscle of COPD patients comprehensively. It has been reported in the literature that the reduction of intercostal and abdominal muscle cross-sectional area in patients with COPD measured by CT was associated with frequent acute exacerbations of COPD (AECOPD) [12, 13]. Therefore, Hyeon Bak et al. used CT to measure the characteristics of the pectoral muscles to predict the severity of the disease [14]. As a result, we chose to study the chest muscles closely related to breathing and lung-function movement, the pectoralis (pectoralis major and pectoralis minor), paravertebral (vertical muscle), diaphragm, intercostal, and serratus anterior. Although CT quantification of the relationship between the chest muscles and COPD has been frequently studied, there is little research on the relationship between chest muscles and COPD exacerbations. In this study, we reviewed inpatient medical records, traced the number of acute exacerbations during the preceding year, measured the chest muscle areas of each patient, and explored the correlation between the two.

## Methods

### Design and patients

**Fig. 1:**
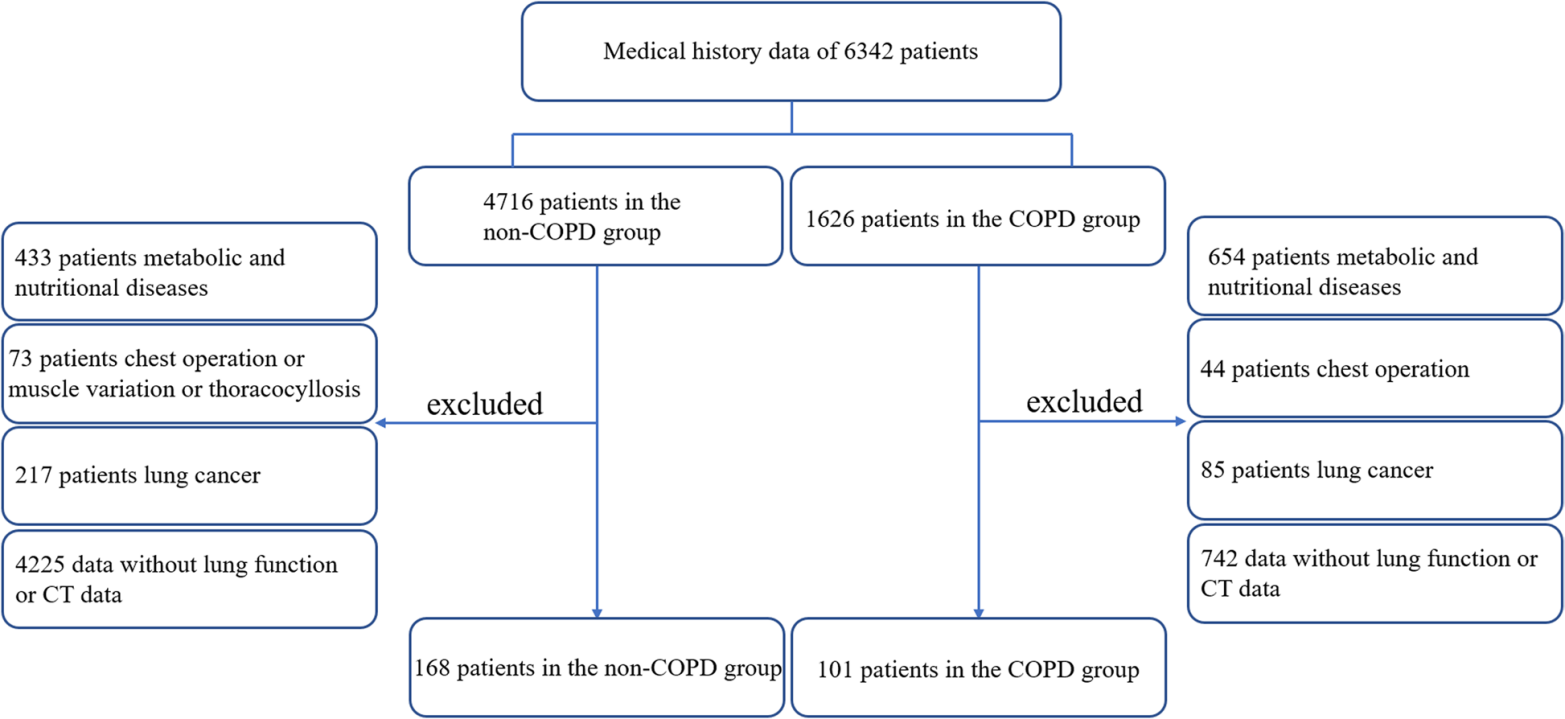
Flow Chart of Study Patients

In this study, we scanned 6342 subjects' electronic records in Guangzhou First People's Hospital from January 2017 to September 2021. For some patients who had been hospitalized repeatedly during this period, we chose the hospitalization records closest in time to September 2021 for data collection. In addition, we gathered the patients’ age, gender, BMI, smoking history, and lung-function-related indicators. There were 168 patients in the non-COPD group and 101 patients in the COPD group. The COPD group inclusion criteria were a clear diagnosis of chronic obstructive pulmonary disease, the presence of acute exacerbations diagnosed per the 2023 Chronic Obstructive Pulmonary Disease Global Initiative diagnostic criteria (a ratio of forced expiratory volume in one second (FEV_1_) to forced vital capacity (FVC) of < 70% after bronchodilator used), [1] complete patient clinical data, CT imaging data, pulmonary function test data, and the ability to have phone consultation return visits about the number of acute COPD exacerbations during the preceding year. A COPD exacerbation is defined as a worsening of a patient's respiratory symptoms that is deemed by the patient's healthcare provider to require systemic corticosteroids, antibiotics, hospitalization, or a combination of treatments [1]. The CT and pulmonary function of the patients were measured during stable COPD. The non-COPD group inclusion criteria were normal chest CT imaging and lung function and complete patient clinical, CT imaging, and pulmonary function test data. Metabolic disease or severe malnutrition have critical effects on skeletal muscles, which may lead to muscle wasting or accumulation. As a result, we excluded patients from the two groups with diseases that impact metabolism and nutritional status (neuromuscular or skeletal muscle diseases), a body-mass index less than 20 kg/m^2^, and patients with malignant tumors, hyperthyroidism, diabetes, and liver or kidney failure. Moreover, patients with a history of chest surgery, thoracic deformities, and muscle variants that cause muscle damage or loss were excluded. A flow schedule was available in Fig. 1. The ethics committee of the First People's Hospital of Guangzhou approved the research protocol.

### CT analysis

**Fig. 2:**
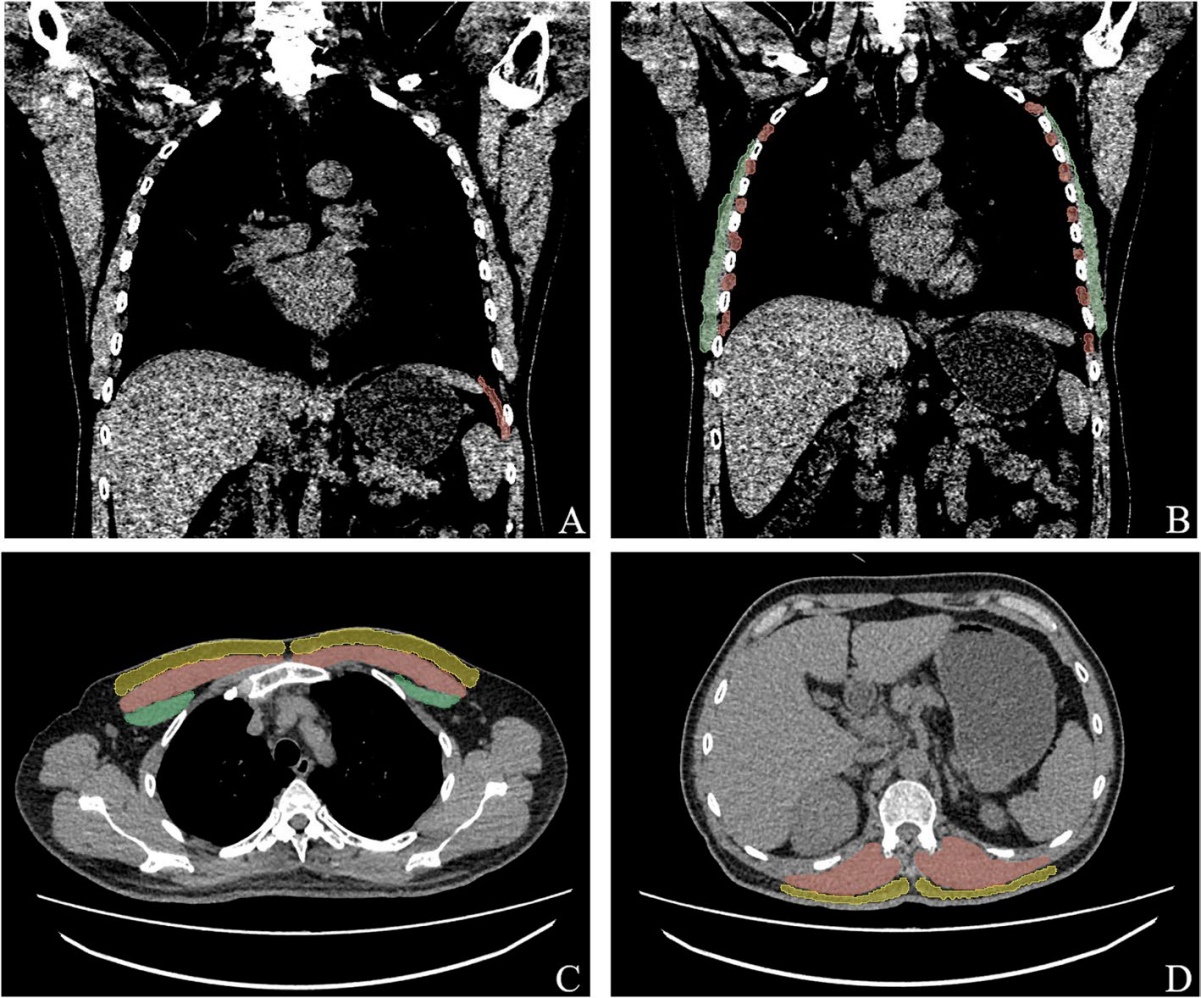
**A** Representative diagrams of a typical measurement methods of diaphragmatic dome (red area). **B** Representative diagrams of a typical measurement methods of musculus serratus anterior (green area) and intercostal muscle (red area). **C** Representative diagrams of a typical measurement methods of pectoralis major (red area), pectoralis minor (green area) and pectoralis subcutaneous fat (yellow area). **D** Representative diagrams of a typical measurement methods of paravertebral muscle (red area) and paravertebral subcutaneous fat (yellow area)

We used the Philips 64 detector row CT imaging system (Philips, Tokyo, Japan). During the CT examination, every patient was placed in the supine position with both upper limbs on top of head. As described previously, measures of lung muscles were performed with the Slicer [15, 16]. The three-dimensional reconstruction of the CT image data was created using 3Dslicer software (version 4.8.1 https://www.slicer.org). The left side of the diaphragm is adjacent to the stomach, spleen, and other hollow organs. In contrast, the right side of the diaphragm is close to the liver and is difficult to distinguish, so the top part of the diaphragm is poorly displayed in imagery. Patterson et al. used CT to examine 102 adults without diaphragmatic lesions, 13 of which showed the right diaphragm, and 82 clearly showed the left diaphragm [16]. Based on the above diaphragm characteristics, we selected the coronal section area of the left diaphragm for measurement. In this area, the inner 2/3 portion of the left diaphragm is poorly displayed; therefore, the measurement was performed on the outer 1/3 portion. We measured the 1/3 portion cross-sectional area on the top surface of the left coronary coronal diaphragm (Fig. 2A, the area in red). Also, we measured the cross-sectional area of the intercostal muscles in the midline horizontal coronal plane from three to eight ribs on both sides. The 1st, 2nd, and 9th to 12th ribs were excluded as it is difficult to distinguish their borders from the chest wall and diaphragm [17]. In addition, the serratus anterior muscle is clearly imaged at this level (measurement of intercostal muscles), so we chose to measure it for the study (Fig. 2B, intercostal muscles area in red, serratus anterior muscle area in green). We measured the cross-sectional area of the pectoralis muscles and fat at the horizontal level in the first slice above the aorta (Fig. 2C, pectoralis major area in red, pectoralis minor area in green and pectoralis subcutaneous fat in orange) and the cross-sectional area of the paravertebral muscles and fat at the horizontal level in the lower slice of the 12th lumbar vertebra (Fig. 2D, paravertebral muscles area in red, paravertebral subcutaneous fat in orange) [18–21]. Emphysema was defined by the percentage of low-attenuation areas below − 950 HU in full-inspiratory CT (inspiratory LAA_-950_).

### Statistical analysis

The primary outcome was the difference in the respiratory muscle areas between the COPD and non-COPD groups. The secondary outcome was the correlation between the respiratory muscles and AECOPD. The continuous variables are normally distributed and represented by mean ± standard deviation. The categorical variables are represented by a percentage. The difference between groups of continuous variables in the normal distribution was determined by t-test or nonparametric test, depending on the data type. The Chi-square test was used for counting variables. We used Pearson correlation analysis to explore the correlation between muscle areas. The ROC curve was used to analyze the relationship between the CT muscle area parameters and COPD and obtain the best cutoff point value for distinguishing COPD. The ward clustering method was used to analyze the COPD and CT muscle area parameters. Univariate and multivariate Poisson regressions were used to analyze the factors related to the number of acute exacerbations during the preceding year. The factors with *P* < 0.2 in the univariate analysis were included in the multivariate analysis. Adjusted gender, age, smoking status, BMI, and COPD classification were used in the multivariate grouping. *P* < 0.05 was considered statistically significant. The cluster analysis was performed using heatmap tools in Hiplot (https://hiplot.com.cn), and other analyses were performed using SPSS software, version 24.0 (SPSS Inc., Chicago, IL, USA).

## Results

### Patient characteristics

We reviewed the medical history data of 6342 subjects who were outpatients or hospitalized in Guangzhou First People's Hospital from January 2017 to September 2021. We selected 269 subjects, with completed data of CT and lung-function tests, according to the inclusion and exclusion criteria described in the Methods (Fig. 1). Among them, the proportion of men was 74.7%, the average age was 68.56 ± 8.86 years old, and the average BMI was 22.79 ± 3.71 kg/m^2^. Of the 269 subjects, 101 had COPD. 85 patients (84.15%) in COPD group were diagnosed emphysema. The diagnosis of emphysema was given by an experienced radiologist and clinician. The demographic and baseline clinical characteristics of the patients in the non-COPD and COPD groups are shown in Table 1. The COPD group had a lower BMI than the non-COPD group (21.14 ± 3.72 kg/m^2^ vs. 23.79 ± 3.35 kg/m^2^, *P* < 0.001) and a higher age (72.37 ± 9.72 years old vs. 66.27 ± 7.44 years old, *P* < 0.001). The two groups had different composition ratios of smoking status (never 52.38% vs. 31.68%, former 24.4% vs. 43.56%, current 23.21% vs. 24.75%, *P* = 0.01). CT imaging index for quantitative evaluation of emphysema was significantly different between the two groups (8.76 ± 9.49% vs. 32.53 ± 16.36%, *P* < 0.001) (Table 1).

**Table 1:** Patient characteristics

	Non-COPD group (*n* = 168)	COPD group (*n* = 101)	*P*-value
Age (yr)	66.27 ± 7.44	72.37 ± 9.72	< 0.001
Male sex (%)	78.57	68.32	0.082
Body-mass index, kg/m^2^	23.79 ± 3.35	21.14 ± 3.72	< 0.001
Smoking status (%)			0.01
Never smoker	52.38	31.68	
Former smoker	24.40	43.56	
Current smoker	23.21	24.75	
Smoking history (pack-yr)	35.86 ± 21.3	39.99 ± 22.74	
Pulmonary function			
FEV_1_- % of predicted value	94.67 ± 18.19	46.35 ± 18.83	< 0.001
FEV_1_:FVC ratio	88.8 ± 9.69	54.86 ± 10.76	< 0.001
COPD stage (%)			-
GOLD I	-	4.95	
GOLD II	-	35.64	
GOLD III	-	38.61	
GOLD IV	-	20.79	
Emphysema (%)	-	84.15	
LAA_950_ (%)	8.76 ± 9.49	32.53 ± 16.36	< 0.001

Plus-minus values are means ± standard deviation

*Abbreviations*: *COPD* Chronic obstructive pulmonary disease, *GOLD* Global Initiative for Chronic Obstructive Lung Disease, *LAA*_*950*_ Low-attenuation area of the lung with attenuation values below − 950 Hounsfield units on inspiratory

### Differences between COPD and Non-COPD respiratory muscles

Compared with the non-COPD group, the muscle areas in the COPD group were smaller: pectoralis major (19.06 ± 5.36 cm^2^ vs. 13.25 ± 3.70 cm^2^, *P* < 0.001), pectoralis minor (6.81 ± 2.03 cm^2^ vs.5.95 ± 1.81 cm^2^, *P* = 0.001), pectoralis subcutaneous fat (25.91 ± 13.23 cm^2^ vs. 18.79 ± 10.81 cm^2^, *P* < 0.001), diaphragmatic dome (1.39 ± 0.97 cm^2^ vs. 0.85 ± 0.72 cm^2^, *P* = 0.011), musculus serratus anterior (28.03 ± 14.95 cm^2^ vs. 16.76 ± 12.69 cm^2^, *P* < 0.001), intercostal muscle (12.36 ± 6.64 cm^2^ vs. 7.15 ± 5.60 cm^2^, *P* < 0.001), paravertebral muscle (14.8 ± 4.35 cm^2^ vs.13.33 ± 4.27 cm^2^, *P* = 0.007), and paravertebral subcutaneous fat (12.57 ± 5.09 cm^2^ vs.10.14 ± 6.94 cm^2^, *P* = 0.001) (Table 2).

**Table 2:** Comparison of the muscle areas

Muscle area	Non-COPD group (*n* = 168)	COPD group (*n* = 101)	*P* value
Pectoralis major area, cm^2^	19.06 ± 5.36	13.25 ± 3.71	< 0.001
Pectoralis minor area, cm^2^	6.81 ± 2.03	5.95 ± 1.81	0.001
Pectoralis subcutaneous fat area, cm^2^	25.91 ± 13.23	18.79 ± 10.81	< 0.001
Diaphragmatic dome area, cm^2^	61.02 ± 22.83	53.61 ± 23.03	< 0.001
Musculus serratus anterior area, cm^2^	28.03 ± 14.95	16.76 ± 12.69	< 0.001
Intercostal muscle area, cm^2^	12.36 ± 6.64	7.15 ± 5.60	< 0.001
Paravertebral muscle area, cm^2^	14.8 ± 4.35	13.33 ± 4.27	0.007
Paravertebral subcutaneous fat area, cm^2^	12.57 ± 5.09	10.14 ± 6.94	0.001

Plus-minus values are means ± standard deviation

**Fig. 3:**
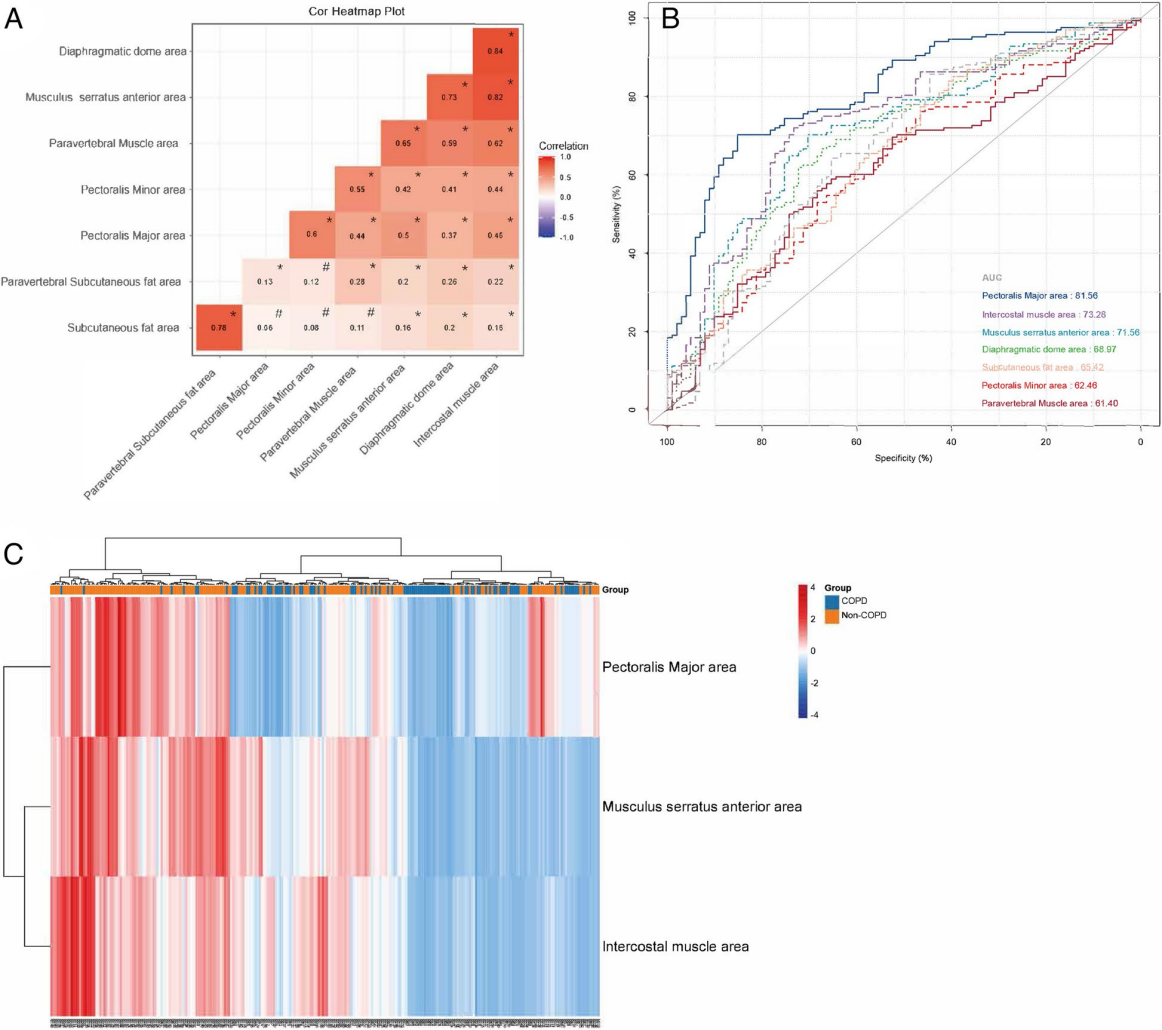
**A** Pearson correlation analysis shown in cor-heatmap plot to explore the correlation between muscle areas, *P-value < 0.05, #*P-value* > 0.05. **B** The ROC curve shows the accuracy of using the chest muscle area to distinguish between patients with COPD and patients who did not have COPD. **C** Cluster analysis heatmap shows three chest muscles (pectoralis major muscle, intercostal muscle, and musculus serratus anterior muscle) expression levels in the COPD and non-COPD groups. Abbreviations: AUC, area under the ROC curve

In analyzing the muscle characteristics of the two groups, we found that the respiratory muscle areas were significantly different. Therefore, we also examined the correlation between each respiratory muscle (Fig. 3A). We found that the correlation coefficient between the diaphragmatic dome and the intercostal muscle areas was 0.84, the correlation coefficient between the musculus serratus anterior and intercostal muscle areas was 0.82, and the correlation coefficient between the musculus serratus anterior and diaphragmatic dome areas was 0.73. The correlation coefficient between the paravertebral subcutaneous fat and the subcutaneous fat areas was 0.78. The ROC curve showed that using the chest muscle area to distinguish between COPD and non-COPD patients was accurate (Fig. 3B). The area under the ROC curve (AUC) of the pectoralis major area was 81.56% (95% CI, 76.4%–86.7%), the AUC of the intercostal muscle was 73.28% (95% CI, 67.1%–79.5%), and the musculus serratus anterior AUC was 71.56% (95% CI, 65.3%–77.9%). We used the pectoralis major, intercostal, and musculus serratus anterior muscles to create a heatmap (Fig. 3C). We then visualized the expression levels in the three COPD and the non-COPD groups’ muscle areas. There were variations between most of the COPD and non-COPD patients, which we used for differentiation. A majority of the patients in the non-COPD group had a high area expression (red), while a small part had a low area expression (blue).

### Factors associated with the number of AECOPD

We used Poisson regression analysis to explore the factors associated with the number of exacerbations during the preceding year in COPD patients. For the univariate regression, we used all the baseline variables to assess the factors related to the number of exacerbations in these populations during the preceding year. Table 3 shows that the smoking status and the musculus serratus anterior area were risk factors for AECOPD during the preceding year. Multivariate Poisson regression analysis shows the pectoralis major area and smoking status were independent factors with the number of patients with AECOPD during the preceding year, controlling for related covariates (gender, age, BMI, smoking status, and COPD stage) (Table 3). The pectoralis major area was an independent predictor of the number of patients with AECOPD after relative risk (RR) adjustment (RR, 0.936; 95% CI, 0.879–0.996; *P* = 0.037). In addition, smoking status (current smoker vs. never smoked) was an independent associated with a higher occureence of the AECOPD after confounding adjustment (RR, 2.392; 95% CI, 1.237–4.627; *P* = 0.01). For each increase of 1 cm^2^ in the pectoralis major area, the risk of acute exacerbations during the preceding year was reduced by 6.8%.

**Table 3:** Univariate and multivariate associations of the number of AECOPD during the preceding year

Variable	Univariate Analysis			Multivariate Analysis		
	RR	95% CI	*P*-value	RR	95% CI	*P*-value
Age	1.015	0.998–1.033	0.087	1.017	0.996–1.038	0.111
Gender	1.354	0.932–1.966	0.112	0.734	0.413–1.304	0.291
BMI	0.994	0.951–1.039	0.783	1.028	0.979–1.08	0.273
Smoking status						
Former smoker vs. never smoked	1.12	0.674–1.860	0.661	1.447	0.743–2.818	0.277
Current smoker vs. never smoked	1.932	1.287–2.901	0.001	2.392	1.237–4.627	0.031
Smoking history (pack-yr)	0.995	0.986–1.004	0.24	0.994	0.985–1.003	0.198
COPD stage (per increase to next stage)	0.999	0.822–1.213	0.991	0.936	0.745–1.177	0.573
Pectoralis major area	0.967	0.923–1.013	0.162	0.936	0.879–0.996	0.037
Musculus serratus anterior area	1.017	1.005–1.029	0.005	1.018	0.998–1.038	0.086
Intercostal muscle area	1.023	0.996–1.051	0.095	1.009	0.961–1.059	0.732

*Abbreviations*: *RR* Relative risk, *95% CI* 95% confidence interval, *COPD* Chronic obstructive pulmonary disease

## Discussion

Although the main pathophysiological changes of COPD are related to lung damage, COPD patients suffer secondary nonrespiratory-related effects of the disease. COPD complications, such as skeletal muscle dysfunction, cause negative symptoms reducing exercise capacity and leading to individual disability [22, 23]. For example, exercise capacity and quadriceps muscle strength are predictors of mortality in COPD patients [24–26], and some experts recommend evaluating them as part of routine clinical examination [27, 28]. Skeletal muscle atrophy is one of the most common extrapulmonary complications of COPD, and studies have shown that muscle wasting could occur in the early stages [1]. In patients with stable COPD, the combined skeletal muscle catabolism and weight loss further aggravate the respiratory symptoms, limit mobility, and affect quality of life, which increases the risk of poor prognosis [29]. For example, current research on COPD has focused on the pathological changes of the airway and lung parenchyma. However, many studies have shown that COPD is closely related to decreased skeletal muscle area and density [30]. Respiratory muscles and auxiliary respiratory muscles are skeletal muscles that play an essential role in developing COPD and should be regarded as important research topics in COPD skeletal muscle wasting. The current study first compared the muscle characteristics of the non-COPD group with the COPD group. All the patients with COPD had different degrees of respiratory muscle area reduction and lower BMIs, suggesting that patients with COPD could have muscle wasting compared with the non-COPD patients. This is consistent with previous reports in the literature. However, it has been reported that there was an ectopic accumulation of fat in patients with COPD [31], and the increase in fat was associated with worsening severity [32]. There seems to be no relevant literature that compares COPD with non-COPD breast fat. As BMI reflects body fat content, it can reflect the amount of subcutaneous fat of the pectoral and paravertebral muscles measured in this study. Therefore, it is possible that the COPD group had less subcutaneous fat in the pectoralis and paravertebral muscles than in the non-COPD group.

After determining that the respiratory muscle area changed, we sought to determine which group of muscles were independent factors in the COPD group and if there was a correlation between the muscles. We used Pearson correlation analysis to explore this. Figure 3A shows the diaphragmatic dome and intercostal muscle areas, the musculus serratus anterior and intercostal muscle areas, and the musculus serratus anterior and diaphragmatic dome areas. The correlation coefficients were all greater than 0.7, suggesting a strong correlation. In contrast, the correlation coefficients between the pectoralis major, pectoralis minor, and other muscle areas were less than 0.6, suggesting a weak correlation. This implies the pectoral muscles were not closely related to the other muscles of the lungs, indicating an independent factor as the difference between the COPD and non-COPD groups. Next, we used the ROC curve to explore which muscle indicators in the COPD and non-COPD groups had reasonable specificity and sensitivity. The results show the AUC of the pectoralis major was 81.56%, the AUC of the intercostal muscle was 73.28%, and the AUC of the musculus serratus anterior was 71.56%. Combining the Fig. 3C heatmap and the above results demonstrated the pectoralis major as the best indicator for evaluating muscle differences between COPD and non-COPD populations.

The deterioration associated with COPD impacts the long-term course of the disease. In a study by Suissa et al. [33], the second severe exacerbation increased the risk of subsequent ones by threefold compared to the first. After the 10th exacerbation, the risk increased by 24-fold. In particular, readmission within 30 days after a COPD exacerbation was associated with an increased risk of death [34]. A patient’s history of exacerbations is the most crucial predictor of frequent ones in the future [35]. Therefore, in studying COPD, it is particularly critical to focus on this aspect. After collecting the relevant patient data, we phone call-recorded the number of acute exacerbations during the year before the CT and lung-function tests. We used the number of occurrences of AECOPD as an independent variable to analyze the related factors. From the multivariate Poisson regression analysis results, the pectoralis major area was an independent factor for the number of AECOPD in patients during the prior year. The pectoralis major area was a strong predictor of the number of AECOPD in patients after RR adjustment (RR, 0.938; 95% CI, 0.88–1; *P* = 0.048). Therefore, our study was valuable to guide the comprehensive evaluation and prognostic analysis of COPD. When evaluating the prognosis of patients with COPD, it is not only necessary to evaluate the lung condition, but also to evaluate extrapulmonary changes, especially the pectoralis muscles.

Our study had some limitations. First, this was a retrospective, single-center study that was not prospective and had a small sample size with insufficient data. Second, a proportion of the patients were females, which eliminated the possibility of correlating muscle area with lung function based on sex because gender differences could have existed in the muscles. As a result, further studies should consist of large samples of follow-up cases. Third, this study is a cross-sectional study, and we cannot determine whether the reduction of pectoral muscles causes acute exacerbation of COPD or the reduction of pectoral muscles caused by acute exacerbation of COPD. We can only do correlation analysis between the above two. Finally, we were unable to measure the pectoralis muscle volume of the participants and could only measure the pectoralis muscle area because of the inaccessibility of the measurement method.

## Conclusion

The pectoralis major muscle area quantified by CT was associated with COPD. In addition, there was a negative correlation between the number of patients who experienced AECOPD during the preceding year and the pectoralis major area. This suggests that the pectoralis major muscle area affected the prognosis of patients with COPD and was an independent risk factor for AECOPD. This result allowed us to evaluate the pectoralis major muscle area using CT in the clinic as a quantitative analysis method to determine the prognosis of patients with COPD, providing novel insights into the treatment and long-term management of COPD.

## Data Availability

The datasets used and/or analyzed during the current study are available from the corresponding author on reasonable request.

## Abbreviations

COPD: Chronic obstructive pulmonary disease
AECOPD: Acute exacerbations of COPD
CT: Computed tomography
ROC: Receiver operating characteristic
AUCs: The areas under the ROC curve
RR: Relative risk
BMI: Body mass index
HGB: Hemoglobin
PaO_2_: Partial pressure of blood oxygen
PaCO_2_: Partial pressure of carbon dioxide
CREA: Creatinine
GOLD: Global Initiative for Chronic Obstructive Lung Disease
CI: Confidence interval
